# Peritoneal full-conditioning reduces postoperative adhesions and pain: a randomised controlled trial in deep endometriosis surgery

**DOI:** 10.1186/1757-2215-6-90

**Published:** 2013-12-11

**Authors:** Philippe R Koninckx, Roberta Corona, Dirk Timmerman, Jasper Verguts, Leila Adamyan

**Affiliations:** 1 Department of Obstetrics and Gynecology, UZ Gasthuisberg, KULeuven, Leuven B-3000, Belgium; 2 Gruppo Italo Belga, Vuilenbos 2, Bierbeek 3360, Belgium; 3 Department of Obstetrics and Gynaecology, VUB, Laarbeeklaan Jette, Brussel 1000, Belgium; 4 Department of Obstetrics and Gynaecology, Jessa Ziekenhuis, Hasselt, Belgium; 5 Department of Reproductive Medicine and Surgery, Moscow State University of Medicine and Dentistry, Moscow, Russia

**Keywords:** Adhesion formation, Full conditioning, N_2_O, Postoperative pain, Postoperative recovery

## Abstract

**Background:**

To translate the concept of full-conditioning (FC) from animal experiments to the human, and to evaluate the efficacy for adhesion prevention. FC consisted of decreasing acute inflammation by 86% CO_2_+ 10% N_2_O + 4% O_2_ for the pneumoperitoneum, cooling of the peritoneal cavity, humidification, heparinized rinsing solution and 5 mg of dexamethasone as demonstrated in animal models.

**Methods:**

A randomized controlled trial (RCT: NCT01344486) comparing standard laparoscopy with full conditioning together with a barrier in a 2/3 ratio in 44 women undergoing deep endometriosis surgery at KULeuven. The primary aim was reduction of adhesions. Secondary aims were CO_2_ resorption, postoperative pain and recovery. Randomization was performed after signing informed consent. Adhesion scoring during second look laparoscopy and pain scoring were done blindly.

**Results:**

In the FC group (n = 16) adhesions were completely prevented in 12/16 women whereas in the control group (n = 11) all women had severe adhesions (P < 0.0005). Also the area, density and severity of adhesions were less. (P <0.001). In the control group, severity, density and area of adhesions were strongly interrelated (P = 0.0001 for all areas) suggesting a common enhancing factor. In the FC group CO_2_ resorption (P < 0.001), postoperative pain (P < 0.001), and CRP concentrations (P < 0.01) were lower while clinical recovery was faster (P < 0.0001) and time to first flatus (P < 0.002) shorter.

**In conclusion:**

This translational research confirms in the human the efficacy of FC in reducing CO_2_ resorption and adhesions with in addition less postoperative pain, lower postoperative CRP concentrations and an accelerated recovery.

## Background

Postoperative adhesions remain a clinical challenge causing chronic pelvic pain, infertility, bowel obstructions [1-3] and reinterventions [4]. This constitutes an economic burden for the health care systems.

Adhesion formation between two opposing lesions is the consequence of a local cascade of events [5,6] comprising a local inflammatory reaction, exudation and fibrin deposition. If fibrinolysis [7] occurs within a few days, the repair mechanism which starts from islands over the entire lesion is completed within 3–5 days irrespective of the surface of the lesion. If the inflammatory reaction and fibrin persist for more than 5 days angiogenesis and fibroblast proliferation result in adhesion formation. Prevention of adhesion formation thus has been based upon keeping surfaces mechanically separated for at least 5 days [8]. In the human this is achieved with resorbable solid or semisolid barriers or with flotation agents. The effectiveness of these products, as demonstrated in small RCT’s since necessitating a repeat laparoscopy, is a reduction of adhesion formation by 40% to 50% for interventions as ovarian cystectomy or myomectomy. For none of these products effectiveness was demonstrated for clinically relevant endpoints as a reduction in chronic pain, infertility or reoperation rate [9,10].

Over the last decade, our group (for review [11,12]) demonstrated in animal models, rabbits and mainly in a laparoscopic mouse model that peritoneal conditioning decreases CO_2_ resorption during surgery [13,14] and the severity of acute inflammation in the entire peritoneal cavity [15] which enhances adhesion formation at surgical trauma sites. This enhancement is mediated by humoral factors in peritoneal fluid since touching small bowels in the upper abdomen [16] can increase adhesions in the lower abdomen. The severity of the acute inflammation increases with mechanical trauma [17] and with the duration and pressure of the CO_2_ pneumoperitoneum [18] (mesothelial hypoxia), by exposure to more than 10% of oxygen [19,20] (through reactive oxygen species, ROS), by desiccation [21] and by blood or fibrin [22]. Beneficial factors are the addition to the pneumoperitoneum of more than 5% of N_2_O [22,23], cooling [24,25] of the peritoneal cavity making cells more resistant to trauma, the addition of dexamethasone [26], and the addition of some 4% of O_2_ to the CO_2_ pneumoperitoneum thus restoring a partial oxygen pressure of 28 mm of Hg [18,27]. In animal models the combination of these beneficial factors, called full-conditioning, decreases adhesion formation by over 80%, N_2_O being the single most effective. If used together with a barrier adhesions decrease by over 90% [26]. This synergistic effect is logic since the mechanisms of action are different. The former decreases acute inflammation in the entire peritoneal cavity while the latter keeps trauma sites separated.

The use of 100% N_2_O instead of 100% CO_2_ for the pneumoperitoneum causes less or no pain during and after surgery as demonstrated under local anesthesia and in prospective [28,29] and in randomized controlled trials [30,31]. The use of N_2_O however never became popular because of the explosion risk with electro surgery at concentrations of N_2_O higher than 30% [32,33]. Since in our laparoscopic mouse model more than 5% of N_2_O had a similar beneficial effect than 100% upon adhesion formation, the mechanism of action has to be a specific N_2_O drug like effect instead of the absence of the irritative effect of CO_2_[22,23].

The goal of the present study was to perform a translational proof of concept trial to investigate the effect of full-conditioning in the human upon CO_2_ resorption during surgery, and upon postoperative pain, adhesion formation and recovery. Given the synergistic effect of full-conditioning and a barrier for adhesion formation a barrier was used at the end of surgery. The results confirm in the human the beneficial effect of full-conditioning.

## Materials and methods

### Full-conditioning in the human

This trial was a translational trial comparing adhesion formation following standard laparoscopy with humidified CO_2_ (Fisher and Pykel humidifier), to full conditioning together with a barrier [26]. The full-conditioning used was based upon the results of adhesion prevention in our animal models and upon the results of cooling the peritoneal cavity in women [34]. A mixture of 86% CO_2_, 10% of N_2_O and 4% of O_2_ was used for the pneumoperitoneum. The peritoneal cavity was cooled, as described, to 30°C by sprinkling 2–3 ml/min of Ringers lactate [34] with 1000 IU of Heparin/L (Leo, Belgium) at room temperature. The gas was humidified using a Fisher & Paykel humidifier (New Zealand) modified by eSaturnus (Leuven, Belgium) in order to deliver to the patient fully humidified gas of 31°C. Upon entrance in the peritoneal cavity some cooling and condensation thus occurred preventing desiccation. The temperature and the relative humidity (RH) of the gas were measured immediately before entering the central trocar and after leaving a secondary trocar [34] in order to control temperature and humidity and the correct functioning of the device. At the end of surgery 5 mg of dexamethasone was administered intramuscularly.

### Surgery

Surgery for deep endometriosis excision was performed as described [35]. Using 3 secondary ports, a CO_2_ laser (Sharplan 1080), sharp dissection and bipolar coagulation, all visible endometriosis was excised. A vaginal or muscularis defect was sutured with monocryl 0 and vicryl 3.0 respectively. The insufflation pressure of the pneumoperitoneum was 15 mm of Hg. Rinsing was performed with Ringers lactate. Continuous aspiration of the pneumoperitoneum through one of the secondary ports was standardized at 2 L/min but was increased temporarily to 25 L/min if necessary for smoke evacuation. In the full-conditioning group care was taken to prevent leaking of gas during surgery and to apply suction for deflation at the end of surgery in order to prevent contamination of the operating theatre with N_2_O.

Surgery itself thus was identical in both groups. We however had the clinical impression of a better image, especially during surgery of long duration with less fogging in the full-conditioning group. Pre and postoperative care was the standard of care of university hospital Gasthuisberg. All women received a mechanical bowel preparation the evening before surgery (Prepakol, Codali, Belgium). They received after surgery 3 L of fluid/day and progressive re-alimentation after first flatus. After a prophylactic bowel suture they received nil by mouth for 4 days. Postoperative pain prevention during the day of surgery and the first postoperative days until flatus was the standard postoperative protocol comprising 3 to 4 IV injections of 1 gr of paracetamol (Perfusalgan, Belgium). Morphine (Dipidolor, Janssens, Belgium) was given only if judged necessary. After first flatus and after starting re-alimentation ibuprofen was given on demand. In order to calculate total pain killer intake 1 administration of morphine, 1 injection of Perfusalgan and 1 tablet of ibuprofen were arbitrarily scored as 4, 2 and 1 respectively.

### Patients and RCT

In this randomized controlled trial standard laparoscopic surgery with humidified CO_2_ at 37°C, was compared to full-conditioning together with a barrier applied at the end of surgery. Hyalobarrier gel (Nordic Pharma) was used in addition to full conditioning since both are synergistic in preventing adhesion formation which was the primary aim of the trial.

The inclusion criterion was a planned laparoscopic excision of a rectovaginal nodule of 1 to 3 cm in diameter as estimated clinically and/or by ultrasound. The presence of adhesions or other endometriotic lesions or previous surgery was not taken into account. Exclusion criteria before surgery were the presence of any immunologic disease or drug intake compromising the postoperative inflammatory reaction, or other clinical problems jeopardizing safety of surgery. Exclusion criteria during surgery, i.e. after randomization, were the absence of a deep endometriosis nodule of 1 to 3 cm in diameter, or a full thickness resection with suturing of the bowel in 2 layers. A full thickness resection was considered an exclusion criterion since safety of barriers has not been demonstrated in this condition.

**Figure 1:**
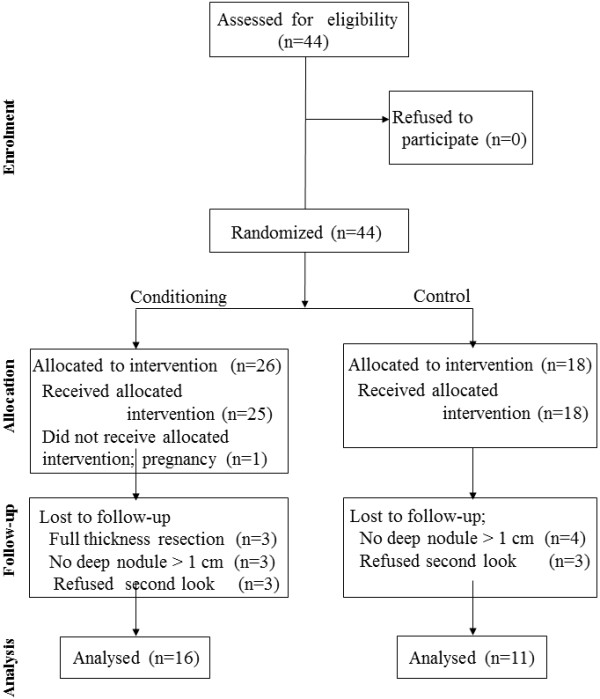
CONSORT statement flow diagram.

This was an investigator initiated trial at University Hospital Gasthuisberg of KULeuven which was IRB approved (S52424) and registered on clintrials.gov nr NCT01344486. After the decision for surgery for deep endometriosis had been taken, the trial was explained. After the patient had signed informed consent, the informed consent together with a request for surgery was sent to the secretary/research nurse. Upon receipt they randomized patients sequentially using sealed envelopes in a 3 to 2 ratio to the full-conditioning group or to the control group. Since the primary aim was the prevention of adhesion formation a 3 to 2 ratio was chosen. Of the 50 patients planned only 44 could be recruited before retirement of the surgeon (PK) from university. Of these 44 women (Figure 1), 26 in the full-conditioning and 18 in the control group respectively, 17 women were drop outs. The reasons were a pregnancy before the intervention (1 conditioning), a full thickness resection (3 conditioning, 0 controls), absence of a large rectovaginal nodule (3 conditioning, 4 controls) while 6 women refused a repeat laparoscopy after the first surgery (3 in both groups).

The primary endpoint of the trial was scoring of adhesions during surgery and during a second look laparoscopy. The secondary endpoints were hemodynamic parameters and CO_2_ resorption during surgery and postoperative pain. The latter was scored 0 to 10 on a visual analog scale the evening of surgery and in the morning of the subsequent 3 days. Pain killer treatment was recorded. Other secondary endpoints were the daily postoperative CRP concentrations, taken in the morning, together with leukocytosis, and temperature.

The RCT was organized as follows. Following randomization, all women scheduled for surgery were entered into our database (with complaints as dysmenorrhea, chronic pain, deep dyspareunia, dyschesia and mictalgia scored 0 to 3 [36]) while immediately after surgery the surgical details [37] (duration of surgery, diameter and depth of each endometriosis localization, rAFS scoring, complications) and the scoring of adhesions were entered. Other data as postoperative complications, VAS scores, and results of biochemical analysis were entered later when available. During the first surgery, the surgeon obviously was not blinded to the treatment allocation. The evaluation of pain after surgery was performed by the registrars or nurses of the department, blinded to the treatment allocation. Second look laparoscopies were scheduled as first intervention in the morning and the patients were anesthetized and draped before the surgeon entered theatre. The surgeon was not informed of the previous treatment allocation. Although this is not a water tight exclusion of bias, recognition of patients in a busy department was close to absent.

### Scoring of adhesions

An early repeat laparoscopy was performed between day 7 and 14 after surgery, since adhesiolysis during early repeat laparoscopy was claimed to be beneficial for the patients [38] and since adhesions are not yet fully organized.

Scoring of adhesions was done separately for 8 sites. Site 1 was the pouch of Douglas with adhesions between the posterior part of the uterus and large or small bowels. Site 2 was the vesico uterine fold i.e. adhesions between the anterior part of the uterus and the bladder or anterior wall. Site 3 and 4 were the left and right ovary and oviducts, i.e. adhesions between ovary and ovarian fossa, between ovary and oviduct, between ovary/oviduct and uterus and between ovary/oviduct and bowel. Site 5 and 6 were the areas between bowels and lateral sidewalls. Site 7 were adhesions from previous surgery between the anterior wall and bowels or omentum and site 8 grouped all other adhesions e.g. adhesions in the upper abdomen. For each site the total cumulative area involved by adhesions was estimated as the diameter (in mm) of the circle when all adhesions in that localization would have been present in one circle. For each site the density and surgical difficulty of adhesiolysis was scored 0 to 3, dense adhesions getting a 3 whereas filmy adhesions were scored as 1. This adhesion scoring was developed from previous scoring systems [39] and reflects the surgery performed on the back side and the anterior side of the uterus, around the adnexa and between bowels and lateral walls. The scoring was remarkably consistent between the surgeon and the assistants (RC, JV) with only minor differences in the estimated total area when the scoring was entered after surgery. In addition a continuous registration of the entire intervention, both the first and the second, were performed as we do routinely for all interventions (Nebula, eSaturnus, Belgium). When during analysis we realized that in the full conditioning group no adhesions were present, blind scoring of the adhesions using the videos was not performed, since absence of adhesions is without bias of judgment.

### CO_2_ resorption and hemodynamic parameters

The anesthetist recorded throughout the intervention every minute blood pressure, temperature, end tidal CO_2_ (mm Hg) and N_2_O, tidal volume (ml) and respiratory frequency (Zeus, Dräger, Germany).

### Statistics

Statistical analysis was done with the SAS program. Power analysis was done with Proc power. Assuming an efficacy of 80%, a large variability of 50%, a total sample size of 20 women already resulted in a power over 99% at 0.01 level. From the original data a series of data were calculated such as severity of adhesion score (area * density), and total adhesion scores (sum of all areas). From the registered diameter and depth of each endometriotic lesion we calculated the volume of deep endometriosis lesions and of the other endometriosis lesions as described [37]. The end tidal CO_2_ concentration, the tidal volume and the respiratory frequency were multiplied as an estimation of the total amount of expired CO_2_. Besides descriptive statistics, statistical significances between groups were calculated using Wilcoxon and Mann–Whitney for non parametric data, Mantel Haenszel for contingency tables and Spearman regression analysis for associations. For analysis of variance (Proc GLM) was used. Mean ± SD are given unless indicated otherwise.

Adhesion scores were analyzed for each localization separately and as a total adhesion score (sum of each individual scores) for area, for density, and for severity (area * density). Specific attention was given to de novo adhesion formation, i.e. adhesions outside the areas of surgery, and to adhesion reformation of adhesions lysed during the first surgery.

## Results

**Figure 2:**
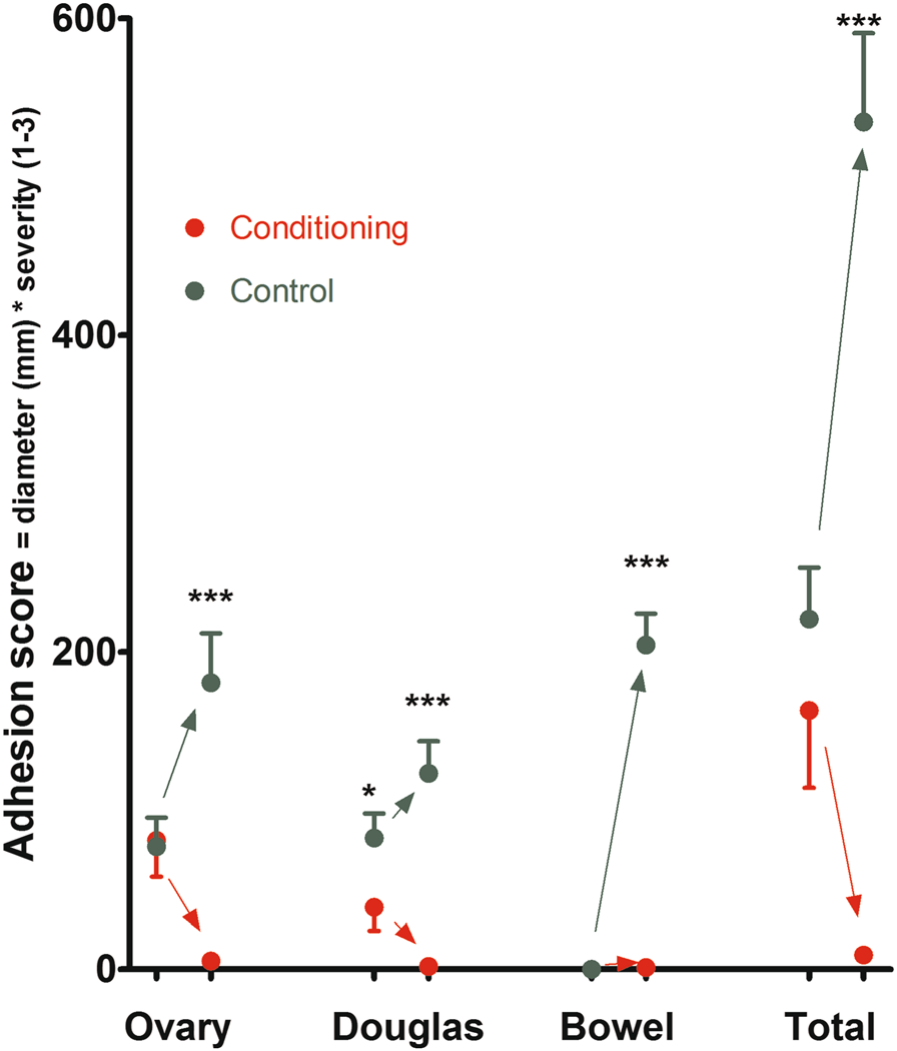
**Adhesion score before surgery and at repeat laparoscopy in the full-conditioning and in the control group, around the ovaries, in the Douglas (between uterus, rectum and recto sigmoid), between bowels and side wall together with the total score (mean and SEM).** Significances are indicated as (*: P < 0.05; ** P < 0.01; *** P < 0.001).

Women in the full-conditioning (n = 16)and in the control (n = 11) group were comparable (NS) for age (33.4 ± 1.6 and 34.2 ± 2.2 years respectively), weight (62.2 ± 2.6 and 69.2 ± 3.4 kgr), height (164 ± 2 and 166 ± 2 cm), dysmenorrhea (2.5 ± 0.3 and 2.0 ± 0.4), deep dyspareunia (1.0 ± 0.4 and 1.0 ± 0.4) and dyschesia (1.3 ± 0.4 and 2.0 ± 0.4). They were comparable (NS) for duration of surgery (141 ± 16 and 101 ± 19 min), estimated blood loss (142 ± 43 and 83 ± 21 ml), diameter of the nodule excised (25 ± 3 and 19 ± 15 mm), calculated volume of the nodule excised (2.0 ± .5 and 1.5 ± .6 cm^3^), presence of cystic ovarian endometriosis (4/16 and 3/11) and rAFS scoring (scored as class I, II, III, IV in 2,4,5,5 and 4,3,2,2 women respectively). Adhesions before surgery were comparable in both groups, except for adhesions between uterus and rectum which were slightly higher in the control group (Figure 2). Since, this could not be explained by a different incidence of adhesions before surgery (11/16 versus 9/11 respectively), by a different incidence of previous interventions (6/16 versus 4/11) nor by the severity or size of the deep endometriosis, nor by the presence or size of cystic ovarian endometriosis, nor by the rAFS score, we consider this as a spurious significance caused by the small groups. No complications or side effects occurred in the control group or in the full-conditioning group.

### Adhesions

At repeat laparoscopy in the control group severe adhesions were found in all women around the ovaries, between the uterus and the rectum or recto sigmoid and between the bowels and the lateral sidewall. In the full-conditioning group only 1 small filmy adhesion was found in 4 women, 12 out of 16 being adhesion free. The differences between full-conditioning and the control group were highly significant whether evaluated as the number of women being adhesion free (P = 0.0005), as the severity score (P < 0.001 for all localizations Figure 2) or as the density and area involved around the left ovary (P = 0.001 and 0.004), the right ovary (P = 0.0003 and 0.0003), between uterus and bowels (P = 0.0002 and 0.0013) and between bowels and side walls (P = 0.0001 and 0.0001).

In the control group the adhesion score after surgery around the ovaries correlated with the adhesion score before surgery around the ovaries (P = 0.002) and between uterus and bowels (P = 0.007). The adhesions after surgery at the different localizations were highly interrelated (total score all P < 0.0001, severity all P < 0.0005), density or area involved all P < 0.001). In the full-conditioning group this could not be evaluated because of the virtual absence of adhesions.

### Postoperative pain and recovery

**Figure 3:**
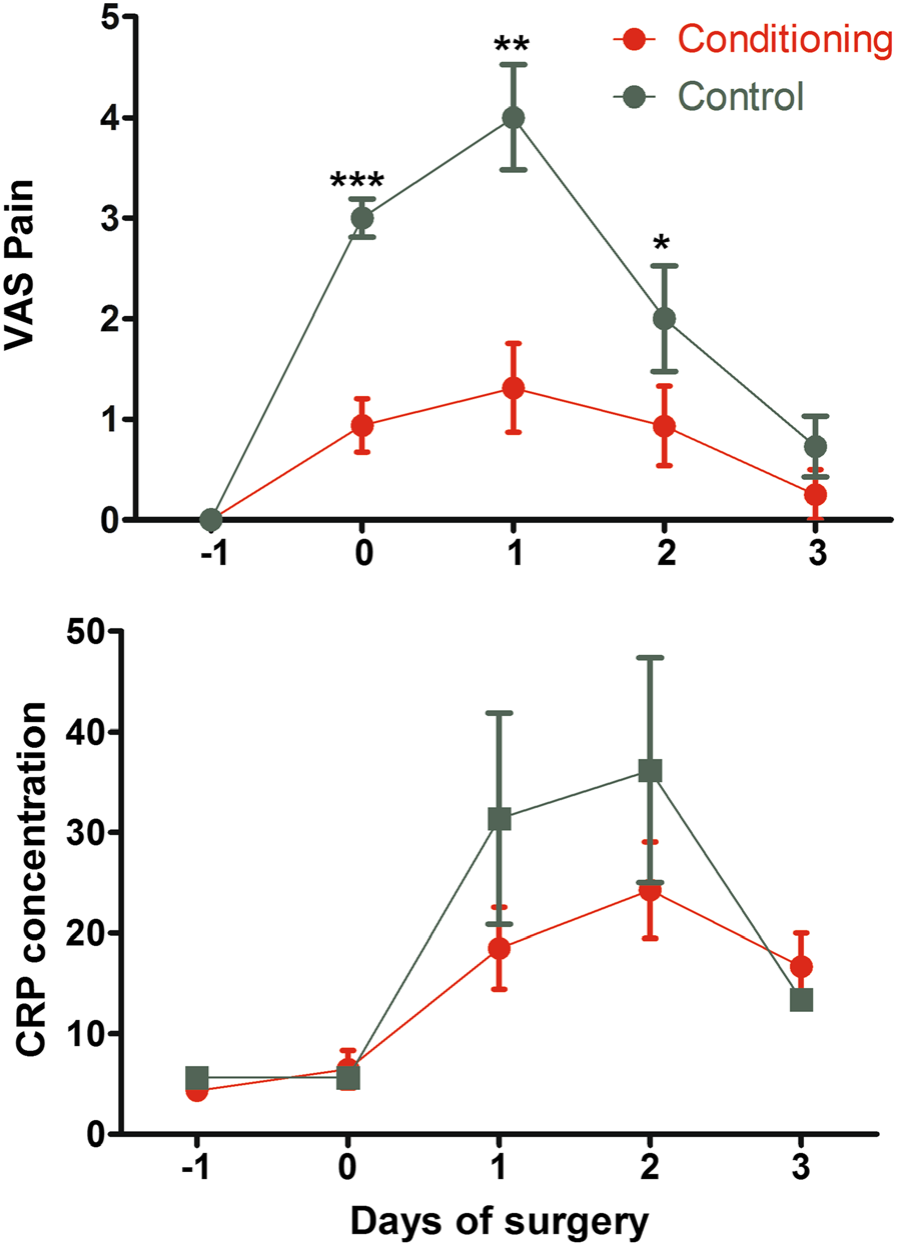
**Pain and CRP concentrations (mean and SEM) in the full-conditioning and in the control group.** Significances are indicated as (*: P < 0.05; ** P < 0.01; *** P < 0.001). The sum of VAS were 3.5 ± 1.1 and 9.7 ± 1.1 (P < 0.001) and the sum of CRP concentrations were 63.5 ± 12.4 and 142.8 ± 19.1 (P = 0.01) respectively.

Postoperative pain scores were much less in the full-conditioning group than in the control group and CRP concentrations were lower (Figure 3), whereas shoulder pain after surgery was not observed in any of the women of the full-conditioning group in contrast to 7/9 in the control group. This decrease in pain and CRP concentrations was reflected in the recovery after surgery but not in the painkiller intake. Recovery following full-conditioning was clinically much faster. Following full-conditioning 9/16 women were mobilized and considered independent the morning after surgery and 7/16 after 48 hours. In the control group all women were still bedridden after 24 hours, while 4 were independent after 48 and 7 after 72 hours (Mantel Haenzel P < 0.0001). The more rapid recovery was also reflected in a shorter time to first flatus being 19.0 ± 2.6 hours and 36.5 ± 3.8 hours respectively (P = 0.002). No differences were seen in time to first stools, being 44.9 ± 5.7 and 57.3 ± 5.0 hours respectively, and in days of hospitalization after surgery. Painkiller intake was not significantly different during the first postoperative days since given routinely. The total pain killer intake on the day of surgery, and on the first second and third postoperative days were 5.7 ± 1.0, 6.3 ± 1.2, 3.7 ± 1.0, 1.4 ± 1.0 and 5.6 ± 0.7, 7.6 ± 1.0, 3.8 ± 0.7, 0.6 ± 0.3 ibuprofen tablets equivalents in the full-conditioning and in the control group respectively. Interestingly Dipidolor was given in 2 women of the control group only. Other parameters as leukocytosis, temperature, or hemogram were comparable in both groups.

### CO_2_ and N20 resorption

**Figure 4:**
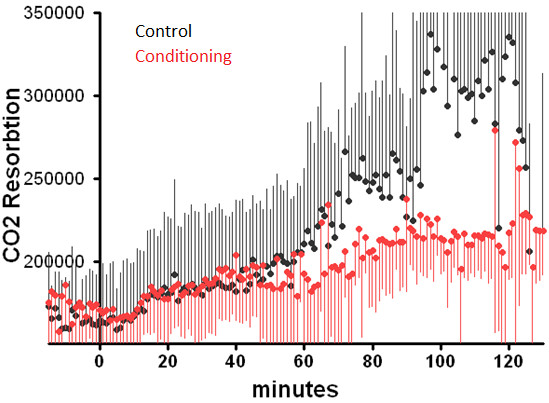
**Resorption of CO**_**2 **_**during surgery in in the full-conditioning and in the control group.** The resorption of CO_2_ is estimated by multiplying the concentration of CO_2_ at the end of expiration (end tidal volume), the tidal volume and the frequency of ventilation.

CO_2_ resorption increased progressively over time in the control group as observed in rabbits (Figure 4). In the full-conditioning group exhaled CO_2_ was significantly less and did not increase substantially after 30 minutes. The hemodynamic parameters did not vary over time. In none of the women receiving conditioning N_2_O could be detected in the expired air.

### Complications and side effects

In none of the women complications or side effects of full-conditioning occurred, including the 3 women with full-conditioning and a full thickness resection and the 3 women who refused a second look laparoscopy.

## Discussion

### Full-conditioning in animal models

From animal models we know that irritation of the large mesothelial cells leads to retraction and bulging, exposing the basal membrane and/or the extracellular matrix, to acute inflammation of the entire peritoneal cavity, to increased CO_2_ resorbtion [13,14] and to enhanced adhesion formation. The severity of this acute inflammation can be decreased by adding more than 5% of N_2_O [22,23] to the CO_2_ pneumoperitoneum, by cooling [24,25] the peritoneal cavity, by preventing desiccation [21], by decreasing blood or fibrin deposition [22] and by the addition of dexamethasone [26]. The addition of some 4% of O_2_ to the CO_2_ pneumoperitoneum, restoring a partial oxygen pressure of 28 mm of Hg [18,27] is beneficial when used alone but has no additive effect when more than 5% N_2_O is used. This acute inflammation of the entire peritoneal cavity was quantitatively the most important cause of adhesion formation. The combination of these beneficial factors was strongly antiadhesiogenic reducing adhesions by 85%. When in addtion a barrier was given adhesions were reduced by over 95%.

### Aim and design of the study

The aim was to perform a translational proof of concept trial to evaluate the concept of full-conditioning in the human. Therefore all factors known to be beneficial in animal models were combined. Since the primary aim was adhesion prevention also a barrier was used. Deep endometriosis surgery was chosen since this is severe surgery of long duration, the severity of mesothelial damage and acute inflammation increasing with time.

The choice of the full-conditioning parameters used was based upon safety and efficacy. Cooling to 30°C by sprinkling 2–3 ml/min of Ringers lactate was demonstrated to be safe [34] and at 30° over 80% of the beneficial effect of cooling is obtained. A concentration of 10% N_2_O was chosen as a compromise between the minimal effective dose of 5% [22,23], and the explosion risk above 30% [32,33]. N_2_O is even safer than CO_2_ since the solubility in water and the lung exchange capacity is even higher [40] and since metabolic consequences of resorption are absent. Safety of N_2_O moreover is ascertained by decades of use in anesthesiology. Although in animal experiments O_2_ did not have an additive effect when 10% of N_2_O was used, we decided to keep 4% of O_2_ since extrapolating animal data to the human. Although oxygen is poorly soluble in water, the risk of gas embolism with 4% is considered extremely low. Heparin in the rinsing solution was used since safe as demonstrated by previous use in the human and since blood or fibrin had such a strong adhesiogenic effect in our mouse model [22,23]. Dexamethasone 5 mg was given since highly effective in our mouse model [26], while being safe as demonstrated in the human.

### Adhesion prevention

The efficacy in preventing adhesion formation and adhesion re-formation by combining full-conditioning with a barrier was nearly 100%. This confirms the observations in the mouse model. The efficacy in preventing adhesion formation is moreover emphasized by the high incidence of adhesion formation in the control group following deep endometriosis excision. The absence of de novo adhesions, as observed in the mouse model was confirmed. This study obviously does not permit to determine the relative importance of each individual factor in the human. From animal data the single most important factors, besides gentle tissue handling, are N_2_O through an unknown drug like effect, and cooling which makes the cells more resistant to trauma by decreasing the metabolism. Humidification, dexamethasone and heparin also contribute to the prevention of adhesions and acute inflammation. The relative importance of each factor moreover is clinically less important since the common mechanism is reducing acute inflammation and since each of them is safe without side effects.

The very strong interrelationship of postoperative adhesions in the control group, around ovaries, between uterus and bowels and between bowels and sidewalls (whether evaluated as total scores or individual density or area) indicates that women with more (severe) adhesions had more (severe) adhesions in each localization. This is consistent with a common factor increasing adhesions at each surgical lesion site, and thus supports the concept of substances from the peritoneal cavity enhancing adhesions. This is not in contradiction with a genetic predisposition for adhesion formation which might be expressed through factors secreted in the peritoneal fluid. Obviously other factors such as fibroblast growth or characteristics cannot be ruled out.

### Postoperative pain

The results are consistent with the hypothesis that 10% of N_2_O is sufficient to reduce postoperative pain as demonstrated when CO_2_ was replaced by N_2_O [28,29,31,41-43]. This hypothesis was derived from the observation that more than 5% N_2_O is sufficient to have a full effect upon adhesion reduction and upon acute inflammation in the mouse model. Indeed postoperative pain was much less and shoulder tip pain was absent [44], recovery was faster and CRP concentrations were lower suggesting a decreased inflammatory reaction in the peritoneal cavity. This decrease in pain was not reflected in the pain killer intake after surgery, since these were given IV according to a fixed hospital protocol until re-alimentation of the patient. It therefore should be stressed that in future experiments pain killer intake should be given on demand. N_2_O is suggested to be the single most important factor in reducing postoperative pain. Since only 10% is effective we have to postulate an hitherto unknown drug like effect of N_2_O. To the best of our knowledge this is the first prevention of postoperative pain. The intraperitoneal administration of local anesthetic drugs after surgery, only reduced pain during the first 6 hours after surgery [45,46]. A major effect of the other factors used besides 10% of N_2_O, is unlikely. Oxygen [47] and dexamethasone [48] were demonstrated to slightly prevent postoperative pain but this effect much less pronounced. Although we cannot exclude an effect of cooling upon pain prevention, laparoscopy under local anesthesia (unpublished observations) demonstrated absence of pain in the absence of cooling.

### CO_2_ resorption

The progressively increasing CO_2_ resorption during surgery and the lower CO_2_ resorption during full-conditioning confirm previous observations in the rabbit model [13,14]. The clinical importance is that occasionally, especially when dissecting the retroperitoneal spaces, CO_2_ resorption can increase so rapidly that the intervention has to be interrupted. We therefore expect that conditioning will permit surgery of longer duration, which can be important in obese patients in steep Trendelenburg.

### Postoperative recovery

Postoperative recovery is faster probably as a consequence of the decreased acute inflammation and the decrease in postoperative pain. It is unlikely that the lower CO_2_ resorption has an important effect upon postoperative recovery, since the metabolic effects of the increased CO_2_ resorption are prevented by the increased ventilation.

### Clinical importance and limitations

The obvious limitation is the small number of patients included in this trial. The strenght is that all observations of this RCT are consistent with the observations made in animal models suggesting similar underlying mechanisms in the human. The clinical importance of full-conditioning during laparoscopic surgery is obvious when considering the efficacy in adhesion prevention, in pain prevention and the accelerated recovery. In addition it can be postulated that also other animal data such as a decreased implantation and metastasis of cancer cells [49], will be true in the human. The latter moreover was already suggested with 100% N_2_O [50]. Moreover it can be speculated that the concept of full-conditioning will be applicable to all cavities lined with a mesothelial cell layer such as in thoracic, cardiac and spine surgery. For cardiac surgery emerging evidence of the beneficial effect of humidification is already available.

## Conclusions

In conclusion, the concept of peritoneal full-conditioning on order to keep the mesothelial cell lining intact and to prevent the postoperative acute inflammation is confirmed in the human. The combination of beneficial factors as demonstrated in animal models is confirmed to be equally effective in the human. Full-conditioning has the potential to become a major step forward in surgery. It does not have side effects and is safe. It decreases CO_2_ resorption during surgery and postoperative pain. It shortens recovery and decreases postoperative adhesions. Besides obvious benefits for the patient it has the potential to decrease health care expenditure by shortening recovery, possibly by a reduced pain killer intake and mainly by the unprecedented effectiveness in adhesion prevention if used together with a barrier. This effectiveness moreover will permit to demonstrate enhanced fertility, decreased chronic pain and reoperation rate. This trial was a first proof of concept trial of adhesion formation in a limited number of patients. The results should be confirmed in other RCT’s. The clinical importance of conditioning should moreover be extended by investigating other anticipated advantages as a lower risk of cancer cell implantation and a lower risk of affecting ovarian reserve during ovarian surgery.

## Competing interests

When the experiments were performed none of the authors had a competing interest. Actually PR Koninckx, after retirement, reports to be stockholder of EndosAT NV.

## Authors’ contribution

All authors were actively involved in the study. PK designed the trial and performed the surgery and the analysis. RC and JV were assisting in the OR and in the follow-up of the patients. DT was responsible for the preoperative ultrasound evaluations. LA (together with KM) made the basic observations on N_2_O and they were closely involved with the design and finalization of the study. All authors read and approved the final manuscript.

## Capsule

Full-peritoneal conditioning strongly reduces postoperative adhesions and postoperative pain and accelerates recovery.
